# Diagnosing and Managing Clinically Silent Lupus Nephritis and Anti-neutrophil Cytoplasmic Antibody-Associated Vasculitis Overlap Syndrome: A Clinical Challange

**DOI:** 10.7759/cureus.24624

**Published:** 2022-04-30

**Authors:** Aqsa Ashraf, Jordan Daloya, Vishal Rana, Aitezaz Ahmed, Alan Kaell

**Affiliations:** 1 Internal Medicine, Mather Hospital, Northwell Health, Port Jefferson, USA; 2 Rheumatology, Mather Hospital, Northwell Health, Port Jefferson, USA

**Keywords:** anti-ds dna, renal pathology, acute renal injury, necrotizing and crescentic glomerulonephritis, lupus nephritis and aav overlap syndrome, antineutrophil cytoplasmic antibody (anca) associated vasculitis (aav), lupus nephritis

## Abstract

Lupus nephritis is typically associated with anti-nuclear antibodies and anti-double-stranded DNA antibodies resulting in the intrarenal immune complex deposition. Levels of anti-double-stranded DNA antibodies reflect disease activity in these patients. With negative anti-double-stranded DNA antibodies, establishing a diagnosis of lupus nephritis is difficult. Lupus nephritis overlapped with anti-neutrophil cytoplasmic antibody-associated vasculitis is both a diagnostic and therapeutic dilemma. Herein, we describe a case of an asymptomatic 41-year-old female who had incidental findings of low hemoglobin and elevated serum creatinine. Making a clinical diagnosis of lupus nephritis and anti-neutrophil cytoplasmic antibody-associated vasculitis in an asymptomatic patient can be challenging and must be made based on the interpretation of evolving serology, imaging studies, and histopathology. Based on extensive workup, the patient was diagnosed with concurrent lupus nephritis and anti-neutrophil cytoplasmic antibody-associated vasculitis overlap syndrome warranting immediate immunosuppressive therapy.

## Introduction

Lupus nephritis (LN) is associated with anti-double-stranded DNA antibody (anti-dsDNA Ab) resulting in intrarenal immune complex (IC) deposition [1]. The prevalence of LN in patients with systemic lupus erythematosus (SLE) has been reported from 15 to 40 per 100,000 patients [2]. Establishing a diagnosis with negative anti-dsDNA Ab is difficult, however, such cases of LN have been reported [3]. More than 100 autoantibodies have been found in patients with SLE [4]. Around 31% of patients with SLE are found to be peri-nuclear anti-neutrophil cytoplasmic antibody (p-ANCA) positive, the prevalence of cytoplasmic (c) ANCA with SLE is extremely rare [5]. These antibodies which are directed against antigens myeloperoxidase-1 (MPO-1) and proteinase-3 (PR-3) are associated with ANCA-associated vasculitis (AAV) which is a type of systemic vasculitis [6]. AAV includes microscopic polyangiitis (MPA), granulomatosis with polyangiitis (GPA; formerly Wegener’s granulomatosis), and eosinophilic granulomatosis with polyangiitis (EGPA; formerly Churg-Strauss syndrome). A literature review indicates around 40 cases of LN overlapped with AAV. P-ANCA and MPO-ANCA were found to be positive in 95% and 82% of cases, respectively [7]. Herein, we describe an unusual presentation of acute renal failure (ARF) with serologic and histopathologic features suggestive of both LN and AAV overlap syndrome.

## Case presentation

An asymptomatic, previously healthy 41-year-old female had routine lab workup when evaluated by a new primary care physician (PCP) that incidentally revealed hemoglobin 6 mg/dL (lower limit of normal {LLN} ≥11.5 mg/dL) and serum creatinine (Cr) 5 mg/dL (upper limit of normal {ULN} ≤1.2 mg/dL). Her PCP informed her of the lab results and referred her to the emergency department (ED) where her severe anemia and renal insufficiency were confirmed. She did recall one day of dark-colored urine without diminished urinary volume two days prior to her ED visit. Medical history was unremarkable except for remote classic migraine and intermittent benign positional vertigo. She recalled having normal routine blood testing two years ago with her previous PCP. Recently, she had a single, unexplained episode of transient, spontaneously resolving lip and eye swelling four weeks before presentation without any associated symptoms. She denied menorrhagia or abnormal bleeding, weight changes, epistaxis, bleeding gums, easy bruising, chest pain, dyspnea, cough, hematochezia, melena, jaundice, changes in bowel habits, focal numbness, weakness or paresthesia, fever, photosensitivity, dry eyes, rashes, oral or nasal ulcers, morning stiffness, joint pain, or swelling. 

On physical examination, the patient was 5ʹ6ʺ tall and weighed 199 pounds, and was in no acute distress. Vital signs included heart rate (HR) 120 bpm, blood pressure (BP) in both arms 202/109 mmHg, oral temperature 99°F (37.2°C), respiratory rate (RR) 18 breathes per minute, and oxygen saturation of 97% on room air. She was alert and oriented and appeared well-nourished with pale conjunctiva. The neurological examination was unremarkable for any cranial nerve, sensory, or motor deficit. The fundoscopic examination was not significant for arteriolar constriction, arteriovenous nicking, flame-shaped hemorrhages, cotton-wool spots, yellow hard exudates, or optic disc edema. The abdominal examination was unremarkable for abdominal distension, tenderness, or organomegaly. No skin rashes, jaundice, or lymphadenopathy were noted. Lungs were clear to auscultation bilaterally. There was normal intensity first and second heart sounds. No murmurs or vascular bruits were audible. The extremities were without any cyanosis or edema and the musculoskeletal examination was insignificant for any joint swelling or tenderness.

Management and further investigation

**Figure 1:**
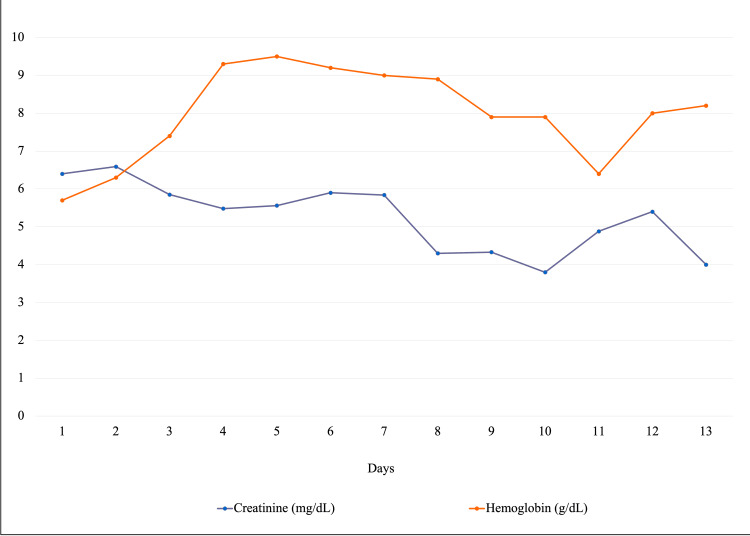
The trend in serum creatinine and hemoglobin levels The image shows marked improvement in creatinine after hemodialysis on day seven.

The patient remained asymptomatic throughout her 13-day hospital course. Laboratory results were as follows: complete blood count (CBC) revealed normocytic, normochromic RBCs without schistocytes or spherocytes with hemoglobin of 5.7 g/dL (LLN ≥11.5 g/dL), hematocrit of 17.8% (LLN ≥34%), normal mean corpuscular volume (87 fL), normal reticulocytes (1.71%), normal leukocytes (6 K/uL), and normal platelets (213 K/uL). The iron panel ruled out iron deficiency with normal serum ferritin (134 ng/mL) and a low total iron-binding capacity of 238 ug/dL (LLN ≥250 ug/dL). Vitamin B12, folate, hemolysis labs (lactate dehydrogenase {LDH}, haptoglobin, bilirubin), and coagulation profile (prothrombin time {PT}, partial thromboplastin time {PTT}) were all within normal limits. Comprehensive metabolic panel included Na+ of 133 mmol/L (LLN ≥136 mmol/L), BUN of 78 mg/dL (ULN ≤20 mg/dL) and Cr of 6.4 mg/dL (ULN ≤1.2 mg/dL); elevated inflammatory markers with an erythrocyte sedimentation rate (ESR) of 90 mm/h (ULN ≤20 mm/h), c-reactive protein of 6.4 mg/dL (ULN ≤5 mg/L), albumin of 3.3 g/dL (LLN ≥3.5 g/dL), and total protein of 10.6 g/dL (ULN ≤8.7 g/dL) without M-spike on serum protein electrophoresis, but polyclonal hypergammaglobulinemia with immunoglobulin (Ig) G of 4991 mg/dL (ULN ≤1660 mg/dL) with normal IgA (395 mg/dL) and IgM (78 mg/dL), immunofixation without any monoclonal band, elevated kappa of 36.69 mg/dL (ULN ≤1.94 mg/dL) and lambda of 21.72 mg/dL (ULN ≤2.63 mg/dL), free light chains along with elevated free light chain ratio of 1.69 (ULN ≤1.65), normal hemoglobin electrophoresis, and positive direct Coombs test. Liver enzymes including aspartate aminotransferase (AST), alanine aminotransferase (ALT), alkaline phosphatase, and indirect bilirubin were within normal limits. Urinalysis revealed pyuria, bacteriuria, hematuria, erythrocytes >50 hpf (ULN ≤5 hpf), proteinuria 100 mg/dL (normal nil), and few hyaline casts. No red blood cell casts were observed. Urine culture showed no growth. Urine electrolytes and analysis indicated intrinsic kidney injury with fractional excretion of sodium 4% (ULN ≤1%), protein creatinine ratio 7:1 (ULN ≤0.2), and 24-hour urine protein in the nephrotic range 5600 mg/24 h (ULN ≤150 mg/24 h). She received two units of packed red blood cells. Infectious workup was negative for human immunodeficiency virus, hepatitis A, B, and C infections. The renal sonogram was unremarkable for nephrolithiasis, hydronephrosis, or renal parenchymal changes and both kidneys were of normal size. Further workup showed positive anti-nuclear antibody (ANA) (screen + at 1:80), negative anti-dsDNA Ab, negative anti-smith antibody, low C3 complement of 74 mg/dL (LLN ≥81 mg/dL), and normal C4 of 16 mg/dL. The patient required hemodialysis and a renal biopsy was obtained. The trend of laboratory results is mentioned in Table 1 and Figure 1.

**Table 1:** The trend of laboratory results WBC: white blood cell

Variables	Reference range	Day 1	Day 3	Day 7	Day 10	Day 13
WBC (K/uL)	4-11	6	6.8	5	6.8	14.9
Hemoglobin (g/dL)	11.5-15.5	5.7	9.3	8.9	7.5	8.2
Platelets (K/uL)	150-450	213	185	70	50	96
Reticulocytes (%)	0.4-2.3	1.71	-	-	1.06	-
Creatinine (mg/dL)	0.7-1.2	6.4	5.85	5.84	3.8	4
BUN (mg/dL)	6-20	78	78	69	23	42
Total protein (g/dL)	6.6-8.7	10.6	10.2	-	-	9.4
Albumin (g/dL)	3.5-5.2	3.3	3.2	-	-	3.2

**Figure 2:**
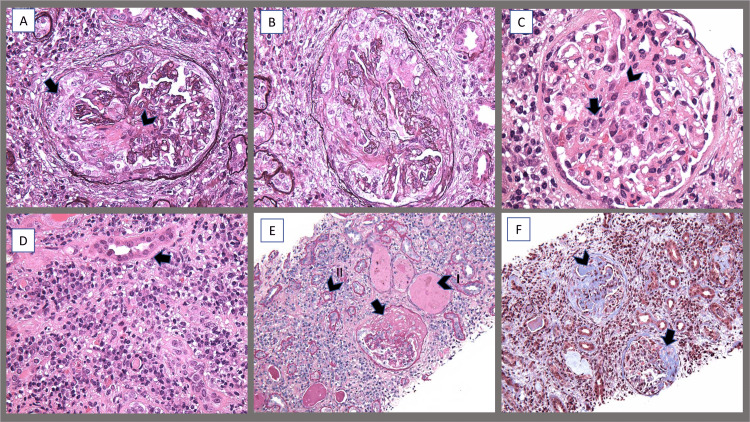
Renal biopsy (light microscopy) images of the patient (A) Crescentic glomerulonephritis with a cellular crescent comprising mesangial and endothelial cells (arrow) with an area of necrosis (arrowhead). (B) Endocapillary and extracapillay proliferative glomerulonephritis. (C) Mesangial proliferation (arrow) with areas of necrosis (arrowhead). (D) Plasma cell-rich inflammation with irregular thickening of tubular basement membrane (arrow). (E) Periodic acid Schiff stain - fibrous crescents (arrow), proteinaceous casts (arrowhead I), thickening of tubular basement membrane (arrowhead II). (F) Trichrome stain - a glomerulus with fibro cellular crescent (arrow), another glomerulus with advanced sclerosis (arrowhead), and background of interstitial inflammation with interstitial fibrosis.

**Figure 3:**
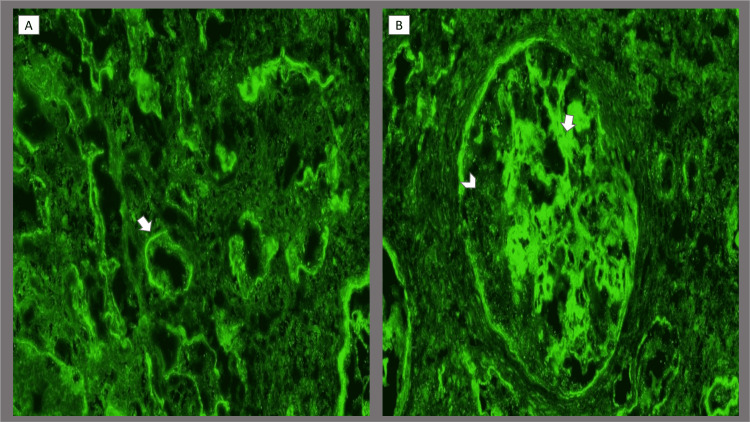
Renal biopsy (immunofluorescence) images of the patient (A) Proximal and distal tubular basement membrane immune complex (IgG) deposition (arrow). (B) IgG immune complex deposition along the glomerular capillary walls (arrow) sparing the crescent (arrowhead).

On post-admission day five, hemolytic anemia was confirmed as evidenced by low haptoglobin of <20 mg/dL (LLN ≥30 mg/dL) and elevated lactic acid dehydrogenase levels 334 U/L (ULN ≤214 U/L). Platelet count dropped to 70 K/uL (LLN ≥150 K/uL). Further testing revealed positive anti-Sjogren syndrome (SS)-A (anti-Ro) antibody of >8 AI (ULN ≤0.9 AI) and B (anti-La) antibody of >8 AI (ULN ≤0.9 AI) and negative anti-glomerular basement membrane antibody. The results of the renal biopsy demonstrated findings consistent with acute and chronic diffuse segmental necrotizing, crescentic proliferative glomerulonephritis with some sclerosis along with extensive immune complex deposition, suggestive of LN class IV. Tubular atrophy, interstitial fibrosis, and tubulointerstitial immune complex deposition were observed (Figures 2A-2F, 3A, 3B).

Following the renal biopsy report, the clinical impression was SLE characterized by features of a positive ANA, low C3, elevated ESR, hemolytic anemia, thrombocytopenia, and biopsy-proven glomerulonephritis consistent with LN. However, a worrisome feature was the presence of active crescentic proliferative glomerulonephritis which could also be indicative of AAV (GPA or MPO). Pending ANCA serology, treatment for LN was initiated with high-dose prednisone (60 mg orally once a day to be maintained for at least four weeks followed by subsequent gradual tapering) and mycophenolate (500 mg orally twice a day with plans for gradual escalation over the next two to three months to a target dose of 1.5 g orally twice a day). Three days after initiation of steroids and immunosuppressive therapy, indirect immunofluorescence (IIF) returned positive for p-ANCA and negative for c-ANCA. Due to the rare co-occurrence of LN and AAV in the same patient, a decision was made to continue the same treatment upon discharge to home and follow up closely with nephrology and rheumatology as an outpatient. Importantly after discharge, the results of a highly positive MPO-ANCA enzyme-linked immunosorbent assay (ELISA) of 43.2 U/mL (ULN ≤9 U/mL) prompted consideration of a change in the steroid-sparing immunosuppressive agent. The positive MPO-1 antibody could be the result of multiple nonspecific autoantibody positivity with no clinical disease features of AAV MPO which can be seen in LN. However, there was a possibility that the positive high-level antibody to MPO, along with crescentic glomerulonephritis, might also be indicative of concomitant AAV. Due to the potentially grave consequences of untreated or inadequately treated AAV, it was decided to add a diagnosis of AAV. In view of the revised diagnosis, induction therapy with rituximab was initiated for AAV. 

## Discussion

This case demonstrates the diagnostic conundrum and therapeutic challenges of LN and AAV overlap syndrome. LN is the renal manifestation of SLE that results from antigen-antibody complexes formed in kidneys in situ rather than passive deposition of circulating immune complexes [1]. On the other hand, AAV is a small vessel involving systemic vasculitis characterized by autoantibodies to PR-3 and MPO-1 [6]. Diagnosing patients with classic symptoms of SLE, LN, and AAV is not difficult, making a clinical diagnosis in an asymptomatic patient with clinical evidence of renal failure can be challenging and must be made based on the interpretation of evolving serology, imaging studies, and histopathology.

Our patient had significant anemia and kidney injury, yet she remained asymptomatic. She was initially diagnosed with LN based on sex, age, presence of hemolytic anemia, thrombocytopenia, elevated creatinine, proteinuria, presence of ANA, and biopsy findings with immune complex rich glomerulonephritis and mesangial proliferation. The glomerular immune complex deposition causes complement activation and leukocyte infiltration. This results in cellular proliferation and release of cytokines, and, in some cases, necrosis, crescent formation, and glomerular scarring [8].

Usually, LN develops within the first three years following the diagnosis of SLE [9]. Testing for anti-dsDNA, anti-C1q autoantibodies, and complement levels (C3 and C4) should be considered in patients with suspected LN [7]. Levels of anti-ds DNA Ab reflect disease presence and activity in patients with LN [10]. Our patient had pathognomonic histopathological findings of LN, yet her anti-ds DNA Ab levels were negative. The absence of anti-ds DNA Ab has a negative predictive value of 91% for active LN [11]. A few cases of LN without anti-ds DNA Ab have been mentioned in the literature [8,12]. The antibody spectrum associated with LN and SLE is not restricted to these antibodies and is extremely diverse. A large variety of autoantibodies have been found in patients with SLE that include Ro/SSA and La/SSB, anti-RNP antibodies, anti-Sm antibodies, anti-nucleosome antibodies, anti-phospholipid antibodies, and anti-NMDAR antibodies [13]. Our patient had positive SSA and SSB antibodies with tubulointerstitial plasma cell infiltrate on renal biopsy. These are findings one would expect to see in Sjogren's syndrome. However, she had no clinical signs of the latter and glomerulonephritis is highly unusual in Sjogren's syndrome, hence, this was ruled out as a probable diagnosis.

In our case, the renal biopsy revealed another interesting finding which is the presence of active crescentic glomerulonephritis which could be indicative of AAV (GPA or MPO). The majority of the patients with AAV have renal involvement, the identification of which if delayed can result in life-threatening end-stage renal disease. Renal involvement in AAV is characterized by rapidly deteriorating renal function and histological features of pauci-immune crescentic glomerulonephritis [14]. The levels of c and p-ANCA correlate with disease activity [15]. 

LN and AAV overlap syndrome is a rare clinical manifestation that is slowly gaining more awareness through an increasing number of documented cases. Due to the rarity of this simultaneous presentation, an epidemiologic pattern has not been well established. It is observed that the overlap of LN and AAV is mostly present in women of child-bearing age who present with severe clinical presentation, including severe renal impairment. Renal biopsy tends to reveal a mixed involvement of immune complex-mediated renal injury, as seen in LN, and evidence of necrotizing and crescentic glomerulonephritis, as seen in AAV [7,16].

Although the underlying mechanism responsible for LN and AAV is not fully understood, presumably it is a type of dysregulation of autoimmunity. The treatment protocols are different for LN and AAV. Hence, the correct diagnosis and timely identification of the responsible disease is important to prevent life-threatening renal dysfunction. Due to the rarity of this overlapping syndrome, an optimal therapeutic approach has yet to be established. The initial induction therapy, apart from high-dose glucocorticoids, is determined by the underlying disease. Immunosuppressive therapy such as mycophenolate and cyclophosphamide are both reasonable options for induction therapy for focal or diffuse LN with similar outcomes in mortality, the incidence of ESRD, and relapse [17]. Rituximab and cyclophosphamide are both used for induction therapy for AAV though authors and physicians tend to favor rituximab over cyclophosphamide as it is found to be less toxic and tolerated well by patients [18]. Further treatment with plasma exchange can be utilized to rapidly remove pathogenic autoantibodies and prevent further deterioration of the kidneys [16].

Our patient initially received treatment with high-dose prednisone and mycophenolate for LN. Later, additional test results were received that showed IIF positive for p-ANCA and negative for c-ANCA with pending PR-3 and MPO-1 confirmatory antibody tests by ELISA. Due to the rare co-occurrence of LN and AAV, it was presumed that in lupus there is nonspecific B cell activation that might lead to hypergammaglobulinemia and possibly to detection of various autoantibodies without associated definite disease other than SLE. The treatment with high-dose prednisone and mycophenolate was continued until additional results were obtained that showed positive MPO-1 antibodies thereby forcing an update of the diagnosis to include AAV, i.e., subtype MPA. In view of the revised diagnosis, induction therapy with rituximab was initiated for AAV.

## Conclusions

A careful assessment of history, physical examination, serology, imaging studies, and histopathological findings should be made to identify the underlying cause of renal dysfunction in patients. Diagnosing and managing connective tissue disorders like LN and AAV-MPA in asymptomatic patients remains a challenge for physicians. Both immunological and pathological data can be helpful in the timely identification of these disorders. Immunosuppressive therapy is warranted in connective tissue disorders; however, induction therapy apart from glucocorticoids is determined by the underlying disease, i.e., mycophenolate mofetil or cyclophosphamide for LN and rituximab or cyclophosphamide for AAV.

## References

[1] Lech M, Anders HJ (2013). The pathogenesis of lupus nephritis. J Am Soc Nephrol.

[2] (2013). Prevalence of systemic lupus erythematosus and lupus nephritis in the United States: analysis of commercial and public insurance billing data. https://acrabstracts.org/abstract/prevalence-of-systemic-lupus-erythematosus-and-lupus-nephritis-in-the-united-states-analysis-of-commercial-and-public-insurance-billing-data/#:~:text=Conclusion%3A%20Based%20on%20an%20analysis,(20%20per%20100%2C000)%20respectively.

[3] Kim HA, Chung JW, Park HJ, Joe DY, Yim HE, Park HS, Suh CH (2009). An antinuclear antibody-negative patient with lupus nephritis. Korean J Intern Med.

[4] Sherer Y, Gorstein A, Fritzler MJ, Shoenfeld Y (2004). Autoantibody explosion in systemic lupus erythematosus: more than 100 different antibodies found in SLE patients. Semin Arthritis Rheum.

[5] Erdoğan O, Oner A, Demircin G, Bülbül M, Memiş L, Uner C, Kiper N (2004). A boy with consecutive development of SLE and Wegener granulomatosis. Pediatr Nephrol.

[6] Kitching AR, Anders HJ, Basu N (2020). ANCA-associated vasculitis. Nat Rev Dis Primers.

[7] Hounoki H, Shinoda K, Matsui A (2021). A case of systemic lupus erythematosus and antineutrophil cytoplasmic antibodies-associated vasculitis overlap syndrome. Case Rep Rheumatol.

[8] Nasr SH, D'Agati VD, Park HR (2008). Necrotizing and crescentic lupus nephritis with antineutrophil cytoplasmic antibody seropositivity. Clin J Am Soc Nephrol.

[9] Korbet SM, Lewis EJ, Schwartz MM, Reichlin M, Evans J, Rohde RD (2000). Factors predictive of outcome in severe lupus nephritis. Am J Kidney Dis.

[10] Isenberg DA, Manson JJ, Ehrenstein MR, Rahman A (2007). Fifty years of anti-ds DNA antibodies: are we approaching journey's end?. Rheumatology (Oxford).

[11] Mok CC, Ho LY, Leung HW, Wong LG (2010). Performance of anti-C1q, antinucleosome, and anti-dsDNA antibodies for detecting concurrent disease activity of systemic lupus erythematosus. Transl Res.

[12] Jain D, Aggarwal HK, Kaverappa V, Dhayia S, Jain P, Yadav S (2014). Anti-dsDNA negative and anti-Ro positive lupus nephritis: a report of a rare case. Reumatismo.

[13] Dema B, Charles N (2016). Autoantibodies in SLE: specificities, isotypes and receptors. Antibodies (Basel).

[14] Sinico RA, Di Toma L, Radice A (2013). Renal involvement in anti-neutrophil cytoplasmic autoantibody associated vasculitis. Autoimmun Rev.

[15] Sowa M, Grossmann K, Knütter I (2014). Simultaneous automated screening and confirmatory testing for vasculitis-specific ANCA. PLoS One.

[16] Xu ZG, Li WL, Wang X (2021). Systemic lupus erythematosus and antineutrophil cytoplasmic antibody-associated vasculitis overlap syndrome in a 77-year-old man: a case report. World J Clin Cases.

[17] Tunnicliffe DJ, Palmer SC, Henderson L (2018). Immunosuppressive treatment for proliferative lupus nephritis. Cochrane Database Syst Rev.

[18] Chung SA, Langford CA, Maz M (2021). 2021 American College of Rheumatology/Vasculitis Foundation guideline for the management of antineutrophil cytoplasmic antibody-associated vasculitis. Arthritis Rheumatol.

